# Editorial: Cerebral and spinal vascular malformations: from bench to bedside

**DOI:** 10.3389/fneur.2023.1235235

**Published:** 2023-08-03

**Authors:** Li Ma, Wuyang Yang, Ethan Winkler, Jan-Karl Burkhardt, Hua Su

**Affiliations:** ^1^Department of Neurosurgery, Beijing Tiantan Hospital, Capital Medical University, Beijing, China; ^2^Department of Neurosurgery, Johns Hopkins University School of Medicine, Baltimore, MD, United States; ^3^Department of Neurological Surgery, University of California, San Francisco, San Francisco, CA, United States; ^4^Department of Neurosurgery, Hospital of the University of Pennsylvania, Philadelphia, PA, United States; ^5^Center for Cerebrovascular Research, University of California, San Francisco, San Francisco, CA, United States; ^6^Department of Anesthesia and Perioperative Care, University of California, San Francisco, San Francisco, CA, United States

**Keywords:** vascular malformation, hemorrhage, dural arteriovenous fistula, moyamoya disease, carotid cavernous fistula (CCF), arteriovenous malformation (AVM), embolization

The advancement of analysis and measurement methods has led to dramatic improvements in genetic sequencing, artificial intelligence, and multi-omics, as well as their integration with clinical data. These groundbreaking methodological advances have ushered in a new era of research in neurosurgery. This era is characterized by the utilization of big data derived from multiple dimensions of neurosurgical diseases. Examples include the analysis of tissue samples at the single-cell or spatially resolved level using multi-omics techniques (1), the application of artificial intelligence to analyze radiological images based on radiomic characteristics, and the design of clinical cohorts or trials with comprehensive biological and imaging databanks. These developments not only provide more opportunities for subgroup analysis to identify specific treatment benefits but also generate data that can be correlated with well-maintained clinical information to unravel causal effects in the pathogenesis of neurological diseases, such as through Mendelian randomization analysis (2). Neurosurgeons, who directly carry out the treatment workflow and acquire tissue samples through surgery, play a crucial role in organizing and interpreting this wealth of big data, which provides a holistic view of the disease and its correlation with clinical information. However, the utilization of advanced research methods in cerebrovascular malformations, characterized by the presence of more mosaicism of both abnormal and normal structures, poses greater challenges in analysis due to the increased presence of background noise, compared to central nervous system malignant tumors. Consequently, concerns arise on how to become a neurosurgeon scientist capable of keeping up with the pace of this new era of research.

The neurosurgeon scientist of the new generation is characterized by three key attributes. Firstly, they possess independent surgical skills that enable them to provide world-class care, which is fundamental for conducting research and contributing to consistent clinical resources and outcomes with an external generalizing value that represents the best patient care. Secondly, they have independent analytical skills that allow them to process and interpret big data, working closely with other researchers who specialize in the analysis of specific types of data. Lastly, they have an independent understanding of the methodological advancements that occur each year, enabling them to conduct research using groundbreaking methods that align with specific research goals, rather than merely serving as conduits for clinical resources. Therefore, the education track of neurosurgeon scientists may need to be extended once again, with enhanced training in surgical skills such as bypass and endovascular surgery, as well as academic training in data science. This could involve pursuing additional academic education either before or after a neurosurgical career and engaging in fellowship training in different neurosurgical specialties.

The aim of this Research Topic is to present advancements across the entire spectrum of central nervous system (CNS) vascular malformations, from bench to bedside. In this collection, we discussed the high-yield controversy on the beneficial effects of embolization for brain arteriovenous malformations (AVMs), particularly with regard to procedure-related hemorrhage and improvements in embolization techniques. Chen et al. reported a severe morbidity rate of 40% following procedure-related hemorrhage in brain AVMs. More than 75% of intraoperative hemorrhages occurred during high-pressure embolysate casting, while 87.5% of postoperative hemorrhages occurred following near-complete embolization. Despite advances in embolization techniques, Lu et al. reported an overall complete obliteration rate of 61% in brain AVMs treated with embolization, which increased to 86% in Spetzler-Martin grade I-II lesions. However, the relatively low obliteration rate compared to surgical resection, coupled with the high risk of unfavorable outcomes following embolization complications in the pursuit of AVM cure, raises questions regarding the careful selection of patients for embolization and the optimal embolization strategies. Recent publications, such as the TOBAS trial (3) and the MATCH cohort (4), do not recommend embolization as the first-line treatment for unruptured brain AVMs. The beneficial effects of embolization in ruptured brain AVMs are also controversial and can vary depending on the embolization strategy employed. Future studies may offer further insights into the selection of patients with ruptured brain AVMs who can truly benefit from safe and curative embolization, as well as the value of adjunctive embolization in high-grade Spetzler-Martin lesions. In contrast to AVMs, embolization has been reported as a feasible treatment strategy for certain rare intracranial fistulas. Tu et al. and Alatzides et al. presented single-center experiences in the management of dural arteriovenous fistulas in the Galen region (Tu et al.) and carotid cavernous fistulas (Alatzides et al.), respectively. These discussions on embolization methods, including transdural venous routes through minimal craniotomy, exemplify a treatment strategy based on a comprehensive understanding of multiple subspecialties. More clinical and basic research are still expected to deconstruct the spinal vascular malformation.

The advancements in imaging analysis have opened up new possibilities for unraveling the intricate hemodynamics and angioarchitecture within cerebrovascular malformations. Quantitative digital subtraction angiography (DSA) has significantly enhanced the measurement and visualization of the imbalanced inflow and outflow, as well as subsequent stasis within brain AVM lesions (5, 6). Radiomics and artificial intelligence (AI) techniques have been employed to depict the detailed morphological features of brain AVMs, holding great promise for substantially altering the paradigm of hemorrhage risk assessment. Within this collection, we also present the progress made in radiographic techniques and analysis methods explored in cerebrovascular research. Hao et al. provide a summary of the applications of functional magnetic resonance imaging (fMRI) and diffusion tensor imaging (DTI) in moyamoya disease. Feng et al., on the other hand, innovatively utilize meta-analysis to investigate the association between aneurysm formation and hypoplasia of the proximal anterior cerebral artery or the presence of a fetal-type posterior cerebral artery. The advancements in imaging techniques and the utilization of automatic analysis based on deep learning algorithms will usher us into a new era of understanding cerebrovascular diseases and the subsequent structural and functional changes in the brain.

During the processing of this topic collection, we observed a diminishing space for research of previous generations in major CNS vascular diseases such as AVMs and aneurysms. Additionally, we identified significant gaps in the understanding of other neurovascular diseases, such as spinal vascular malformations, dural arteriovenous fistulas, and carotid cavernous fistulas. It is crucial to encourage open-access publications that allow leading scientists to disseminate state-of-the-art understanding, methodologies, and techniques as public educational resources, correcting the misconception that open-access publication is a repository for less recognized research findings. The policies regarding processing charges for open-access publications should also be improved to create an equal platform for investigators in early-career stages or from developing countries. By making fundamental learning resources for the new era of research widely available, we hope to gradually transform the learning curve for the new generation of neurosurgeon scientists.

## Author contributions

LM prepared the editorial. All authors contributed to the article and approved the submitted version.
